# E3 Ligase FBXW7 Facilitates *Mycobacterium* Immune Evasion by Modulating TNF-α Expression

**DOI:** 10.3389/fcimb.2022.851197

**Published:** 2022-05-16

**Authors:** Jingrui Song, Jin Chao, Xiaohong Hu, Xin Wen, Cairong Ding, Dan Li, Ding Zhang, Shanshan Han, Xiang Yu, Bo Yan, Zhu Jin, Yinhong Song, Jacqueline Gonzales, Laura E. Via, Lu Zhang, Decheng Wang

**Affiliations:** ^1^ Hubei Key Laboratory of Tumor Microenvironment and Immunotherapy, China Three Gorges University, Yichang, China; ^2^ Institute of Infection and Inflammation, China Three Gorges University, Yichang, China; ^3^ Medical College, China Three Gorges University, Yichang, China; ^4^ Department of Tuberculosis, The Third People’s Hospital of Yichang, Yichang, China; ^5^ Department of Pathology, Yichang Central People’s Hospital, Yichang, China; ^6^ Shanghai Public Health Clinical Center, Fudan University, Shanghai, China; ^7^ Tuberculosis Research Section, Laboratory of Clinical Immunology and Microbiology, and Tuberculosis Imaging Program, Division of Intramural Research, National Institute of Allergy and Infectious Diseases, National Institutes of Health, Bethesda, MD, United States; ^8^ Institute of Infectious Disease and Molecular Medicine, University of Cape Town, Cape Town, South Africa; ^9^ Engineering Research Center of Gene Technology, Ministry of Education, Department of Microbiology, School of Life Science, Fudan University, Shanghai, China

**Keywords:** FBXW7, mycobacteria, granuloma, TNF-α, ubiquitination

## Abstract

Tumor necrosis factor alpha (TNF-α) is a crucial factor in the control of *Mycobacterium tuberculosis* (*Mtb*) infection. Pathogenic mycobacteria can inhibit and/or regulate host cell TNF-α production in a variety of ways to evade antituberculosis (anti-TB) immunity as well as facilitate immune escape. However, the mechanisms by which TNF-α expression in host cells is modulated to the benefit of mycobacteria is still an interesting topic and needs further study. Here, we report that macrophages infected with *Mycobacterium marinum* (*Mm*)—a close relative of *Mtb*—upregulated the expression of E3 ubiquitin ligase FBXW7. Specific silencing FBXW7 with small interfering RNA (siRNA) significantly elevates TNF-α expression and eventually promotes the elimination of intracellular bacteria. In turn, overexpression of FBXW7 in Raw264.7 macrophages markedly decreased TNF-α production. Furthermore, partial inhibition of FBXW7 in an *Mm*-infected murine model significantly reduced TNF-α tissue content, alleviated tissue damage as well as reduced the bacterial load of mouse tails. Finally, FBXW7 could decrease TNF-α in a K63-linked ubiquitin signaling dependent manner. Taken together, our study uncovered a previously unknown role of FBXW7 in regulating TNF-α dynamics during mycobacterial infection, which provides new insights into understanding the role of FBXW7 in anti-tuberculosis immunity and its related clinical significance.

## Introduction

Tuberculosis (TB), caused by *Mycobacterium tuberculosis* (*Mtb*), is still a serious disease that results in significant morbidity and mortality worldwide in humans and animals (Cicchese et al., 2018). Over thousands of years of co-existence, *Mtb* has evolved multiple strategies to evade control by the host. Tubercular granulomas are the battlefields resulting from the *Mtb*-host interaction, and their ability to contain the *Mtb* organisms determines the outcome of the primary infection (Khan et al., 2019). Macrophages are the primary immune cells that bacilli encounter. They phagocytose *Mtb* in the naive stage of infection, and that phagocytosis leads to an early inflammatory response. Macrophages play a central role in granuloma development during early infection and granuloma maintenance during latency through the recruitment of other diverse cell types to the site of infection (Khan et al., 2019). They produce a variety of cytokines after activation, such as tumor necrosis factor α (TNF-α), interferon (IFN-γ), IL-6, IL-12 (Reed et al., 2004; Bhatt and Salgame, 2007; Bernut et al., 2016). As a pleiotropic cytokine, TNF-α plays a multifaceted role during *Mtb* infection including attracting immune cells to the site of infection through a cascade reaction (Dorhoi and Kaufmann, 2014) and maintaining granulomas to prevent *Mtb* from spreading out from the diseased tissue (Flynn et al., 1995; Clay et al., 2008; Dorhoi and Kaufmann, 2014). It has been found that pathogenic mycobacteria strains induced lower TNF-α than non-pathogenic ones (Beltan et al., 2000; Yadav et al., 2004; Dorhoi and Kaufmann, 2014; Olsen et al., 2016), suggesting that negative regulation of TNF-α is beneficial to the survival of *Mtb* (Elsaidi and Lowary, 2014). As a pathogenic mycobacteria species, *Mtb* has developed a series of strategies to regulate TNF-α secretion in order to facilitate its immune escape and eventually favor its survival (Stanley et al., 2003; Reed et al., 2004; Yadav et al., 2004; Kurtz et al., 2006; Elsaidi and Lowary, 2014; Wang et al., 2015; Olsen et al., 2016; Yao et al., 2018; Wang L. et al., 2020).

The protein F-box and WD repeat domain containing 7 (FBXW7) is a subunit of an E3 ubiquitin ligase and is considered to have a tumor suppressor function. FBXW7 recognizes numerous oncoproteins like c-Myc, Notch, c-Jun, Cyclin E, and MCL1 as substrates in order to promote their degradation *via* the ubiquitination pathway (Yumimoto and Nakayama, 2020). FBXW7 mutation can lead to its substrate accumulation and subsequently promotes the occurrence of malignant tumors (Refai et al., 2018). Recently, the role of FBXW7 in infectious diseases has attracted the attention of researchers. It has been reported that adenovirus (Isobe et al., 2009), respiratory syncytial virus (RSV) (Song et al., 2017), human papillomavirus (HPV) (Gillison et al., 2019), and prion infection (Xu et al., 2016) aberrantly regulate host FBXW7 to facilitate their transmission and dissemination. FBXW7 expression was reported to be decreased in RSV-infected patients, resulting in the accumulation of SHP2 to further degrade the expression of retinoic acid-inducing gene I (RIG-I) in the immune response, allowing RSV to escape host recognition (Song et al., 2017). In contrast, FBXW7 was reported to be up-regulated in prion diseases, which led to the ubiquitination and degradation of mTOR and acceleration of autophagy possibly as a prion control strategy (Xu et al., 2016). Chen reported that ectopic expression of FBXW7 targets the nonstructural protein 5B (NS5B) of HCV and that ubiquitination mediates degradation of NS5B thus inhibiting viral replication (Chen et al., 2016). Furthermore, recent research into the *Ehrlichia chaffeensis* TRP120 HECT E3 Ub ligase showed that it could target FBXW7 as a substrate to promote *E. chaffeensis* infection *via* maintaining the stability of Notch, c-Jun, and c -Myc and supporting cell survival (Wang J. Y. et al., 2020). Recently, a genome-wide association study identified that FBXW7 was significantly downregulated in the chicken after infection with *Salmonella pullorum*, suggesting it may also play a role in controlling *S. pullorum* infection (Li et al., 2019). Moreover, two studies of FBXW7’s function in macrophages suggested that FBXW7 played crucial roles in macrophage-pathogen interaction (Song et al., 2017; Wang J. Y. et al., 2020). These findings highlight the crucial functions of FBXW7 as a ubiquitin ligase in the pathogenesis of a variety of infectious agents. However, the role of FBXW7 in the pathogenesis of bacteria and the details of its molecular and immunological mechanisms have not been described. With the above findings as background, we hypothesized that FBXW7 plays a role in granuloma formation by modulating the pro-inflammatory responses in host macrophages during *Mtb* infection.

In the present study, we found that FBXW7 was highly expressed in Raw264.7 macrophages infected with *Mycobacterium marinum* (*Mm*), a close relative of *Mtb* that induces caseating granulomas in the mouse tail, similar to those formed in human TB (Carlsson et al., 2010). In Raw264.7 cells, specific knockdown of murine FBXW7 by small interfering (si) RNA effectively enhanced TNF-α production as well as inhibited expression of anti-inflammatory cytokines TGF-β and IL-10. In contrast, FBXW7 overexpression inhibited TNF-α secretion and promoted the production of TGF-β and IL-10. Moreover, partially inhibiting FBXW7 in *Mm*-infected mice not only decreased TNF-α production *in vivo* but also markedly relieved infection-related tissue damage. FBXW7 was found to mediate TNF-α degradation *via* K63-linked ubiquitination. Our data demonstrated that FBXW7 played a crucial role in the formation of *Mtb*-induced granulomas by altering the expression of cytokines especially the level of TNF-α for the mycobacterial survival in the host.

## Materials and Methods

### Bacterial Strains and Growth Conditions

The *Mycobacterium marinum* (*Mm*) M strain (ATCC BAA-535) was originally obtained from Dr. L. Ramakrishnan (University of Washington, Seattle) and preserved by Dr. Gao (Fudan University); it was used as the wild-type (WT) parental strain in this study. *Mm* induces granulomas in the tail rather than the organs of the mouse because the tail has a lower temperature that is more permissive to *Mm* growth than its body (Carlsson et al., 2010). A previously generated phthiocerol dimycocerosates (PDIM)- deficient mutant derived from *Mm* M strain (fadD26:Tn) was used as the attenuated strain (Yu et al., 2011). The WT and △PDIM strains were labeled respectively by Wasabi and *td*Tomato fluorescence according to the published protocols for △RD-1 strain (Takaki et al., 2013). All *Mm* strains were grown at 32°C in Middlebrook 7H9 broth (Difco) supplemented with 10% oleic acid albumin dextrose-catalase (OADC enrichment), 0.5% glycerol, and 0.05% Tween 80 or on Middlebrook 7H10 agar supplemented with 10% OADC and 0.5% glycerol. For fluorescence-labeled strains, 100 μL of 50 mg/mL hygromycin B was added to 7H9 OADC per 100 mL.

For the detection of bacterial load in tail tissues, tail tissues with lengths of 5 mm were weighed and homogenized in 0.5 mL of DMED medium containing 0.1% Triton-X. The homogenized tissue suspension was diluted and plated on Middlebrook 7H10 agar with 10% OADC and 0.5% glycerol at 32°C for 8 to 10 days. Then, the number of bacteria colonies was counted, and the bacteria load was expressed as colony forming units (CFU)/g.

### Mice and Reagents

C57BL/6 mice were obtained from the Center of Experimental Animals of China Three Gorge University (CTGU). All animal experiments were reviewed and approved by the Animal Care and Use Committee of CTGU. Raw264.7 macrophages (ATCC, TIB-71) were maintained in DMEM medium, Thioglycolate GIBCO BRL (Grand Island, NY, USA) with 10% FCS.

Antibodies to the haemagglutinin tag (Ha; sc-805, 1:1000), β-actin (sc-47778, 1:1000) and FBXW7 (sc-293423, 1:200) were from Santa Cruz, Inc. Myc tag (16286-1-AP, 1:1000) and Flag tag (20543-1-AP, 1:1000) were from Proteintech Group, Inc. Alexa Fluor 488-labeled anti-rabbit (A27034, 1:1000), Alexa Fluor 647-labeled anti-mouse IgG (A28181, 1:1000) and Alexa Fluor Plus 405-labeled anti-mouse (A48255, 1:1000) were from Thermo Fisher Scientific. Antibodies specific for FBXW7 (ab109617, 1:500), TNF-α (ab255275, 1:1000), TGF-β (ab215715, 1:1000) and IL-10 (ab271261, 1:1000) were from Abcam. Antibody specific for NF-κBp65 (8242, 1:1000) was from Cell Signaling Technology. MG132 (M8699) was from Sigma-Aldrich.

### Plasmid Constructs and Transfection

Recombinant vector encoding mouse TNF-α (NM_013693.3; NM_001278601.1) was created with PCR-based amplification of Raw264.7 complementary DNA, and then subcloned into the pcDNA3.1 eukaryotic expression vector (Invitrogen). The vectors were constructed by OBiO Technology (Shanghai) Corp., Ltd and were confirmed by DNA sequencing. The Myc-FBXW7, HA-Ub, HA-K48R Ub, and HA-K63R Ub constructs were kindly provided by Prof. Qingqing Wang (Zhejiang University School of Medicine, Hangzhou, China). All constructs were also confirmed by DNA sequencing. The plasmids were transfected into Raw264.7 with ExFect Transfection Reagent (#T101-01, Vazyme, Nanjing, China) according to the standard protocol.

To confirm whether the degradation of TNF-α was mediated by FBXW7 with ubiquitination, Raw264.7 cells were transfected with Flag-TNF-α, Flag-TNF-α, and Ha-Ub, or Flag-TNF-α, Ha-Ub and Myc-FBXW7 plasmids for 24 h. The cells were then treated with MG132 (25 mM) for 6 h before cell harvest and applied with immunoblotting as described in section 2.6.

To determine the type of FBXW7-mediated ubiquitination, we transfected Flag-TNF-α, cotransfected with Flag-TNF-α and Ha-Ub plasmids, co-transfected with Flag-TNF-α plasmids, Ha-Ub and different doses of Myc-FBXW7 plasmids, and co-transfected with Flag-TNF-α, Myc-FBXW7, and different Ha-Ub mutants (K48R or K63R) plasmids into Raw264.7 cells, respectively.

### *Mm* Infection

Macrophages were seeded with a density of 6*10^5^ cells per well into six-well plates. The cells were allowed to adhere to the plate overnight and infected with *Mm* strains (WT or △PDIM, MOI = 5) or treated with the same volume of DMEM medium as if infected (MOCK). The cells were then incubated at 37°C in 5% CO2 for 1 to 12 h. The cells with different treatments were harvested at the specified times and were used for *q*RT-PCR or Western blot Assays. Three replicate wells were used for each time point (n=3).

**Figure 1:**
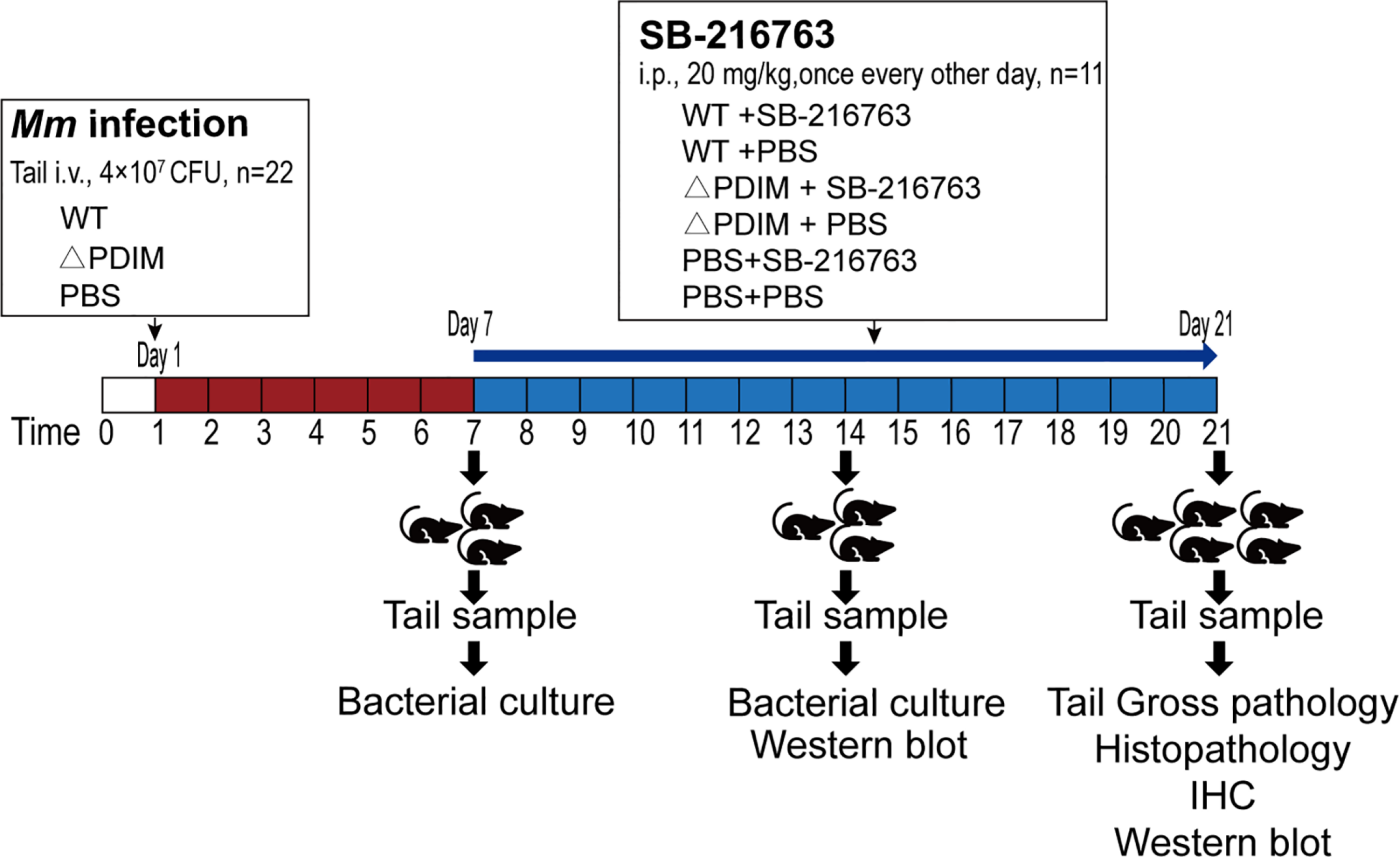
Design and protocol for the animal experiment. Sixty-six C57BL/6 mice were divided randomly into three groups. Twenty-two animals were administered PBS by i.v. to the tail vein. Twenty-two were injected with WT *Mm* while another twenty-two received △PDIM. After 7 days, the animals were divided randomly into two groups of eleven animals each. These groups were administered SB-216763 or PBS by i.p. every other day until sacrifice at 21 days. On days 7 and 14, three mice for each group were sacrificed, and then tails from them were sampled for detection of bacterial load and Western blot assays. On day 21, all the mice were sacrificed for assays. IHC, Immunohistochemistry staining.

For mice (**Figure 1**), SPF, aged (6-8 weeks old)- and sex-matched groups of littermate mice were infected with 4*10^7^ CFU *Mm via* tail intravenous injection (n=22/group). On the seventh day post infection, half mice from each infection were intraperitoneally injected with SB-216763 (Selleck Chemicals, Houston, TX) with a dosage of 20 mg/kg (PBS with 5% DMSO) in a volume of 150 μL once every other day, until the 21^st^ day when the observation period was ended. The dosage was according to the instruction of the manufacturer (Park et al., 2014). The tails of the mice were observed for 3 weeks for granuloma formation. On days 7 and 14, three mice for each group were sacrificed, and then tails were sampled for detection of bacterial load and Western blot assays. On day 21, all the remaining mice were sacrificed.

### Quantitative Reverse Transcriptase PCR

Cells or tissues were immediately treated with Trizol reagent (Invitrogen) and total RNA was isolated from the cells or tissues according to the manufacturer’s instructions. Then, single-strand cDNA was generated from total RNA by using reverse transcriptase (Takara). Quantitative real-time reverse transcriptase PCR (*q*RT-PCR) analysis was applied with SYBR Green Master Mix (#Q111-02; Vazyme) according to the manufacturer’s instructions. Delta Ct value (ΔCt = Ct _target genes_ – Ct _house keeping genes_) was estimated, then ΔΔCt value was calculated as (ΔCt _infected group_ - ΔCt _mock-infected group_), and the relative expression was expressed as 2^(-ΔΔCt)^. The sequences for primers are listed in **Supplementary Table 1**.

### Western Blot Assays

For tail tissues from mice, about 0.2 g of tissue was homogenized and lysed with cell lysis buffer (#P0013B, Beyotime Biotechnology, Shanghai, China) containing a protease inhibitor “cocktail” (#P8340, Sigma); The harvested cells were lysed with cell lysis buffer with a protease inhibitor “cocktail”. After centrifugation at 12,000 g for 5 min at 4°C, the protein concentrations were measured with a BCA assay (#AR1189, Boster Biological Technology, Wuhan, China). Then, the extracts were boiled for 10 min in SDS-PAGE Sample Loading Buffer (#P0015L, Beyotime Biotechnology). Equal amounts of protein extract were separated by SDS–PAGE, then transferred onto PVDF blotting membrane (#10600023, GE Healthcare Life Science, Little Chalfont, Buckinghamshire, UK), blocked with 5% BSA or 5% defatted milk in TBST buffer (10 mM Tris/HCl pH 7.4, 75 mM NaCl, 1mM EDTA, 0.1% Tween-20), probed with the antibody for immunoblot analysis, and incubated with secondary antibody. The secondary antibodies used were either HRP-conjugated anti-mouse or anti-rabbit IgG depending on the primary antibodies. Immunoreactive bands were visualized by detection of chemiluminescence with ChemiDocT XRS+ Molecular Imager (Bio-Rad, Hercules, CA, USA), and quantified by analysis with the Image Lab software (Bio-Rad).

### Histopathology Examination

Tails sections from control or infected mice were fixed in 10% phosphate-buffered formalin and embedded into paraffin. Paraffin embedded lung tissues from patients were collected by the Third People’s Hospital of Yichang and provided by the hospital. The patients were diagnosed as clinical TB according to a previous report (Franco et al., 2017). *Mtb* experimentally-infected rabbits (*Mtb* strain HN-878) and mice (*Mtb* strain H37Rv) paraffin embedded lung sections were kindly provided by Clifton E. Barry, Tuberculosis Research Section, Laboratory of Clinical Immunology and Microbiology, NIAID, NIH. All the tissues were sectioned, stained with hematoxylin and eosin (H & E) solution, and examined by light microscopy.

### Immunohistochemistry (IHC) Staining

Sections of lungs and tails were analyzed by immunohistochemistry assay as previously described (Wang et al., 2012). In this assay, the primary antibody is FBXW7 antibody from rabbit (dilution of 1:400, Abcam) or mouse (dilution of 1:200, Santa Cruz), and the secondary antibody is the HRP-conjugated goat anti-rabbit IgG (dilution of 1:200) (BM3894; Boster Biological Technology co. ltd).

### Immunofluorescence Staining

Macrophages were cultured on sterilized glass coverslips in 24-well plates and infected with *Mm* for 4 h or treated as if infected (MOCK), as the method mentioned in section 2.4. Cells were fixed with 2% paraformaldehyde for 30 min at 4°C, permeabilized with 0.1% Triton X-100 for 15 min, blocked with 5% BSA in TBST for 30 min, and stained with rabbit anti-FBXW7 antibody and mouse anti-NF-κBp65. Following the primary antibody, the samples were stained with FITC-labeled anti-rabbit secondary antibody and Cy3-labeled anti-mouse secondary antibody. DAPI (4, 6-diamidino-2-phenylindole; Sigma) was used to stain the nuclei. The co-localization of FBXW7 and NF-κBp65 was detected by imaging with a Nikon AIR^+^ confocal laser microscope. The signal for nuclear p65 was determined by counting the number of nuclei (DAPI) merged with p65, dividing by the total number of nuclei scored. At least 200 total nuclei per well were counted in each of the triplicate wells.

### Statistical Analysis

Differences between the groups in the ordinal data were analyzed by the Mann-Whitney U test to compare two data sets or Kruskal-Wallis one-way analysis of variance (ANOVA) to compare more than two data sets. The statistical analyses were performed using GraphPad Prism 4.0 for Windows (GraphPad Software, La Jolla, CA, USA). Statistical significance was considered at *P*-value less than 0.05.

## Results

### FBXW7 Expression in *Mm*-Infected Macrophages and Granulomas of *Mtb*-Infected Humans and Animals

**Figure 2:**
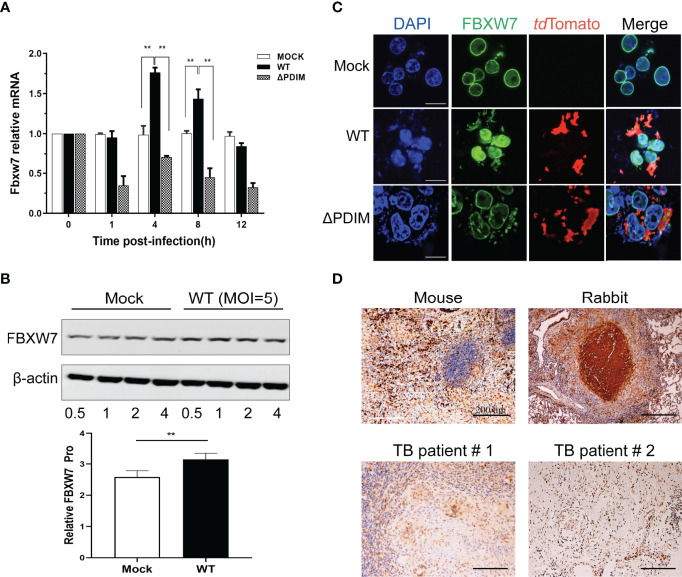
FBXW7 is differently expressed in macrophages after WT *Mm* infection and in the lung tissues of hosts with *Mtb* infection. **(A)** mRNA expression of FBXW7 in macrophages after *Mm* infection (MOI of 5) over 12 hours (n=3) was detected by *q*RT-PCR. Increased expression of FBXW7 mRNA was observed at 4 and 8 h in cells post infection with WT *Mm* relative to MOCK (*P* < 0.01). **(B)** protein expression of FBXW7 in macrophages after WT *Mm* infection (MOI of 5) over 0.5 to 4 h relative to ß-actin. **P < 0.01 **(C)** Representative images of macrophages stained for FBXW7 (green) and the *Mm* marker *td*Tomato (red). Nuclei were counterstained with DAPI to identify the cellular location of FBXW7. Scale bar, 10 μm. **(D)** Representative immunohistochemical images of FBXW7 (brown precipitate) in the granulomas of lung samples from experimentally *Mtb*-infected C57BL/6J mouse and NZW rabbit and two natural Mtb-infected patients. Nuclei are counterstained with hematoxylin. Scale bar, 200 μm. The experiments were performed three times with similar results.

To investigate whether FBXW7 was regulated in WT_*Mm*-infected Raw264.7 macrophages, we infected Raw264.7 macrophages with WT_*Mm*, harvested the macrophages at various times post infection, and analyzed FBXW7 mRNA and protein levels. FBXW7 mRNA was found to be increased in WT_*Mm* infected macrophages at 4 h and 8 h post infection (hpi), compared to MOCK and △PDIM cells, *P* < 0.01 (**Figure 2A**). FBXW7 protein content was also higher than MOCK at 4 h, *P* < 0.01 (**Figure 2B**). FBXW7 was mainly expressed in the karyotheca both in the MOCK and the △PDIM groups, but highly expressed in the whole nucleus of *Mm* infected cells (**Figure 2C**). To verify this result, we further detected the FBXW7 in lung granulomas of TB patients and lungs of *Mtb*-infected mice and rabbits. Immunohistochemistry stain demonstrated that FBXW7 was extensively expressed in the multinuclear giant cells of the granulomas of rabbits and humans and the multinuclear giant cells in the regions with inflammatory cells infiltrating those of the mouse (**Figure 2D**). Taken together, these results revealed that FBXW7 was highly upregulated in WT_*Mm*-infected macrophages and present in granulomas.

### FBXW7 Altered the Secretion Profile of Inflammatory Mediators During Mycobacterium Infection

**Figure 3:**
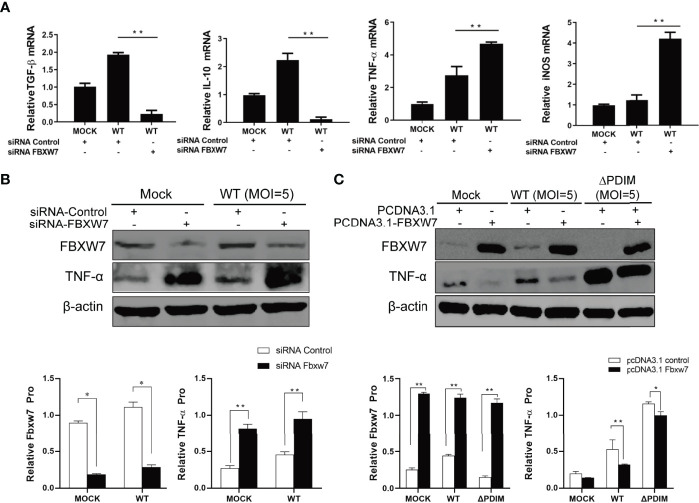
FBXW7 influences the expression of inflammatory mediators in *Mm* infection. **(A, B)** Raw264.7 cells were transfected with FBXW7 mimic (siRNA-Control) or FBXW7 inhibitor (siRNA-FBXW7) for 24 h, which was followed by infection with Mm at 5 MOI for 4 h or mock infection treatment. The expression of pro- and anti-inflammatory mediators were detected by *q*RT-PCR **(A)** and Western blot **(B)**. TGF-ß and IL-10 mRNA expression were down-regulated by FBXW7 silencing in *Mm* infected cells. TNF-α mRNA and protein and iNOS mRNA expression were up-regulated by FBXW7 silencing in *Mm* infected cells. **(C)** Raw264.7 cells were transfected with PCDNA3.1 or PCDNA3.1_FBXW7 for 24 h, which was followed by infection with wild type *Mm* or △PDIM strains at 5 MOI for 4 h. The expression of TNF-α was detected by Western blot. β-actin was used as an internal control. All the data in the histograms represent the mean ± standard deviation SD. *P < 0.05 and **P < 0.01. All the experiments were repeated three times with similar results.

To investigate the role of FBXW7 in host immune response, we silenced FBXW7 with small interfering RNA (siRNA) in Raw264.7 cells (**Supplementary Figure 1A**) and found the anti-inflammatory cytokines TGF-β and IL-10 mRNA expression were significantly decreased in the FBXW7-knockdown cells infected with *Mm* (*P* < 0.01, **Figure 3A**), whereas expression of pro-inflammatory cytokine tumor necrosis factor alpha (TNF-α) and inducible nitric oxide synthase (iNOS) were significantly enhanced (*P* < 0.01, **Figure 3A**). The analogous change was detected on the protein level of TNF-α (*P* < 0.01, **Figure 3B**) but not TGF-β and IL-10 (**Supplementary Figure 1B**). Further, when we over-expressed FBXW7 with CRISPR/Cas9 in Raw264.7 cells (**Supplementary Figure 2**), we found that overexpression of FBXW7 led to decreased TNF-α protein expression both in WT (*P* < 0.01) and △PDIM cells (*P* < 0.05, **Figure 3C**).

### FBXW7 Depressed the Activation of NF-κB p65

**Figure 4:**
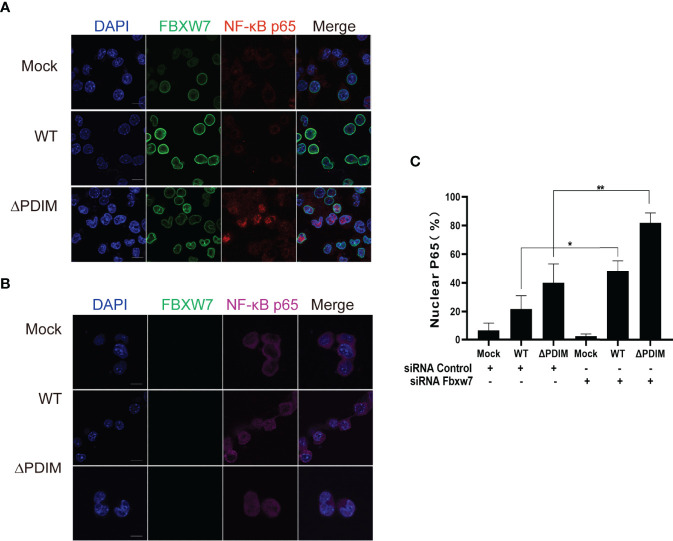
FBXW7 influences the expression and location of NF-κBp65 in *Mm*-infected macrophages. **(A, B)** Representative immunofluorescence images of macrophages stained for the FBXW7 (green, FITC-labeled second antibody) and the NF-κBp65 (red or purple, Alexa Fluor 647- or Alexa Fluor Plus 405- labeled the second antibody). Nuclei were counterstained with DAPI to identify the cellular location of FBXW7 and NF-κBp65. Scale bar, 10 μm. **(C)** the percentage of nuclear NF-κBp65 positive cells in the immunohistochemical images in **(A, B)**. The averaged values represent the mean ± standard deviation (SD) (n = 3 per group). At least 200 total nuclei per well were counted, and the signal for nuclear p65 was quantified as the percentage of nuclei number (DAPI) merged with p65 divided by the total number of nuclei counted per well, n = 3 wells per condition. *P < 0.05 and **P < 0.01.

NF-κB is a well-known nuclear transcription factor, and its activation affects the expression of proinflammatory cytokines such as TNF-α, IL-6, and chemokines, leading to the inflammatory response (Yu et al., 2020). To investigate whether FBXW7 regulates the NF-κB signaling pathway, we utilized an immunofluorescent assay to observe the interaction between FBXW7 and NF-κBp65 along with *Mm* infection. After being infected with wild type *Mm*, the fluorescently labeled expression of NF-κBp65 was inhibited in the Raw264.7 cells (**Figures 4A, C**), and significantly enhanced in the FBXW7-silenced cells (*P* < 0.05, **Figures 4B, C**). Meanwhile, the expression of NF-κBp65 translocated into the nucleus from the cytoplasm in *Mm*-infected cells when FBXW7 was silenced (**Figure 4B**). However, co-localization between FBXW7 and NF-κBp65 was not observed during the response to *Mm* infection (**Figure 4A**).

### Partial Inhibition of FBXW7 Alleviated Tail Injury in Mice Following *Mm* Infection

**Figure 5:**
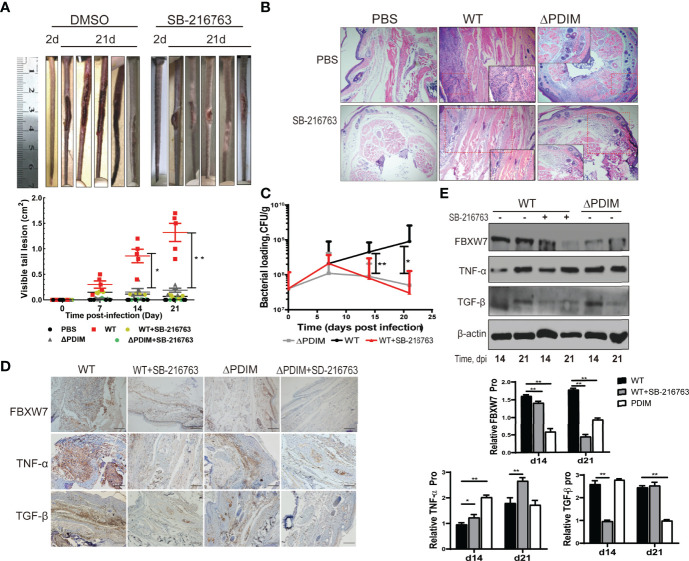
Inhibition of FBXW7 by SB-216763 alleviated tail injury in mice following *Mm* infection. **(A)** Tails of WT *Mm* infected mice treated with vehicle or SB-216763 for 14 days. The area of all visible lesions on each tail was combined on the 21^st^ day post-infection and graphed; the bars represent the mean ± standard deviation (SD) (n = 5 per group; 5 groups shown in legend). Significant differences between the WT and WT+SB-21676 are indicated (*P < 0.05; **P < 0.01); **(B)** Representative images of H&E staining of tail lesions. More lymphohistiocytic infiltrates were observed in the tail tissues infected with WT *Mm* compared to the PBS group, while these were somewhat alleviated when the mice were treated with SB-216763. Magnification = 4×. **(C)** Bacterial burden in the whole tail was quantified by enumeration of CFU at multiple time points post-infection. The averaged values represent the mean ± standard deviation (SD) (n = 3). **(D)** Representative immunohistochemical images of FBXW7 staining (brown precipitate) in the tail samples from mice treated as indicated. Nuclei are counterstained with hematoxylin. Scale bar, 500 μm. **(E)** The expression of TNF-α and TGF-β were detected by Western blot in the tail samples from mice at 14 and 21 days post-infection. β-actin was used as an internal control. *P < 0.05 and **P < 0.01.

To investigate the function of FBXW7 in the formation of host granulomas induced by *Mm in vivo*, we infected mice with WT or the △PDIM *Mm* strain (4*10^7^ CFU) *via* tail vein injection. Seven days post infection (dpi), intraperitoneal injection with SB-216763 to inhibit the FBXW7 was initiated. At 21 dpi, mice with WT *Mm* infection displayed severe tail damage (**Figures 5A, B**) with lymphocyte infiltration in the tail tissues compared to ones in the uninfected and △PDIM groups, *P* < 0.05. The *Mm*-induced damage in the tails was alleviated when the mice were treated with SB-216763 (**Figures 5A, B**). Moreover, the bacterial cultivation showed a significantly decreasing bacterial replication in the tail tissues of SB-216763-treated mice compared to those given vehicle alone, (*P* < 0.05, **Figure 5C**).

FBXW7 expression in the tail tissues was evaluated with IHC. We found that WT *Mm* strain infection increased FBXW7 expression in the tails of mice while △PDIM strain infection did not (**Figure 5D**). The effects of FBXW7 were further evaluated on the protein expression of cytokines including TNF-α and TGF-β in the tail tissues with Western blot. Consistent with the results in the cell infection model, mice infected with WT *Mm* strain expressed more FBXW7 expression both at 14^th^ dpi and 21^st^ dpi (*P* < 0.01), lower TNF-α expression at 14^th^ dpi (*P* < 0.01) and lower TGF-β expression at the 21^st^ dpi (*P* < 0.01) compared to the △PDIM group (**Figures 5D, E**), respectively. When the FBXW7 expression was inhibited by SB216763 treatment (*P* < 0.01, **Figures 5D, E**), mice in the WT *Mm* group expressed more TNF-α (*P* < 0.05) in the tail tissues than untreated ones at the 14^th^ and 21^st^ dpi (**Figures 5D, E**). Meanwhile, TGF-β expression was inhibited in tails of the WT *Mm* group at 14^th^ (*P* < 0.01) but not at 21^st^ dpi (**Figures 5D, E**).

### FBXW7 Facilitates the Degradation of TNF-α by K63-Linked Ubiquitylation

**Figure 6:**
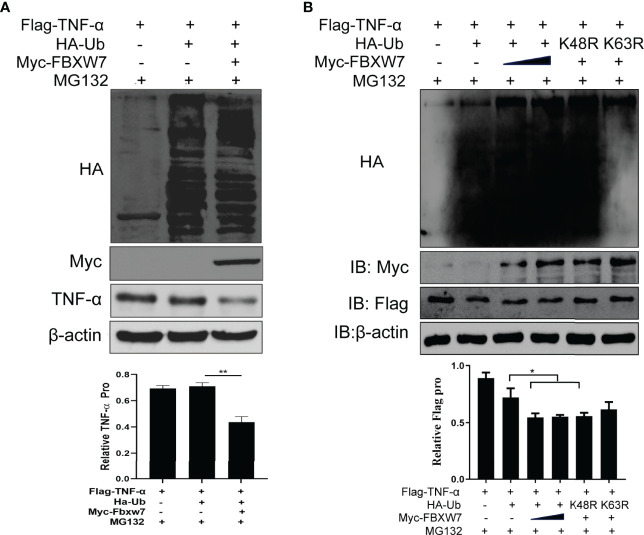
FBXW7 facilitates the degradation of TNF-α by K63-linked ubiquitylation. **(A)** Immunoblot of Raw264.7 cells co-transfected for 48 h with Myc-FBXW7, plus Flag-TNF-α, HA-ub and treated with MG132 before cell harvest; adding FBXW7 to the system significantly decreases TNF-α (*P* = 0.01) **(B)** Immunoblot of Raw264.7 cells co-transfected with Flag-TNF-α, Myc-FBXW7 along with Ha-Ub, the mutant ubiquitin Ha-K48R Ub or Ha-K63R Ub and treated with MG132 before cell harvest. Similarly, the addition of myc-FBXW7 and K48R mutant Ha-Ub reduced the expression of Flag-TNF-α significantly (*P* = 0.05). The experiments were performed three times with similar results. *P < 0.05 and **P < 0.01.

We transfected FBXW7 and Ub plasmids into Raw264.7 cells and found that FBXW7 mediated the degradation of TNF-α in the presence of Ub (*P* < 0.01, **Figure 6A**). In order to confirm this result, we co-transfected FBXW7 with Ub K63 or K48 mutant plasmids and found that FBXW7-mediated ubiquitination was abolished when the cells were transfected with K63-mutant Ub plasmids, but not in cells receiving K48-mutant Ub plasmids (**Figure 6B**). Similarly, the degradation of TNF-α was also found in macrophages transfected with the K48-mutant but not K63-mutant Ub plasmids (*P* < 0.05, **Figure 6B**). These data indicate that FBXW7 mediated degradation of TNF-α is dependent on K63-linked polyubiquitination.

## Discussion

Ubiquitination is crucial posttranslational modification controlling many cellular processes and functions in the immune response (Jiang and Chen, 2011). It has been reported that *Mtb* adopted a diplomatic strategy on the ubiquitination of the host to benefit its persistent intracellular infection through restricting host inflammatory responses (Wang et al., 2015; Wang L. et al., 2020). In the past decade, as an E3 ligase, FBXW7 has been found to play numerous roles in cancers including angiogenesis, tumor initiation and progression, proliferation, and differentiation (Yumimoto and Nakayama, 2020). Recently, FBXW7 has also been shown to have multiple functions in virus infection *via* interacting with diverse host molecules such as SHP2 (Song et al., 2017), mTOR (Xu et al., 2016), and NS5B (Chen et al., 2016) in immune responses. Although a previous study reported that FBXW7 was downregulated after *Salmonella* infection (Li et al., 2019), the function of FBXW7 in the immune responses during bacterial infection is still unknown.

In this work, we use *Mm* as a model for *Mtb* in the vertebrate host. *Mm* is a close genetic relative of the *M. tuberculosis* complex, so it is an ideal model species to study the mechanisms of mycobacterial pathogenesis (Tobin and Ramakrishnan, 2008). However, as a natural pathogen of ectotherms, *Mm* does not colonize mammalian internal organs productively because its optimum growth temperature is lower than their internal body temperature (Carlsson et al., 2010). Fortunately, besides forming granulomas on zebrafish (Pozos and Ramakrishnan, 2004), *Mm* also induces caseating granulomas, similar to those formed in human *tuberculosis*, in the mouse tail because it is cooler than their bodies (Carlsson et al., 2010). Therefore, we have used Mm induced tail injury model in mice to begin to explore the role of FBXW7 in *Mtb*-host interactions. PDIM is an essential lipid component of the normal cell wall of *Mtb*. It is also an important virulence factor that plays a vital role in the pathogenesis of *Mtb* (Arbues et al., 2014; Quigley et al., 2017). PDIM-deficient *Mtb* have significantly weakened virulence compared to PDIM-replete wild-type strains in mouse infection models (Quigley et al., 2017). Here, we use a PDIM-deficient *Mm* as the virulence-attenuated control in comparison to the more virulent WT *Mm*.

In this study, we found that the FBXW7 expression was increased in macrophages after infection with *Mm*. To confirm the result *in vivo*, we also found FBXW7 expression in the granulomas of lung tissues from humans and animals infected with *Mtb*. FBXW7 promoted anti-inflammatory cytokines including TGF-β and IL-10 and inhibited the pro-inflammatory cytokine such as TNF-α indicating that FBXW7 played a suppressive role in the host’s inflammatory responses to *mycobacterium* infection. Meanwhile, the partial inhibition of FBXW7 with SB-216763 alleviated injuries to tissues and decreased the bacterial load of WT *Mm*, implying that FBXW7 may also enhance the pathogenic process by mycobacteria. This is the first report describing the function of FBXW7 during *Mtb* infection.

Developing granulomas are dynamic, which is closely related to the phenotypes of macrophages (Khan et al., 2019). *Mtb*-induced granulomas have been shown to contain two types of macrophages including classically activated macrophages (pro-inflammatory or M1 macrophages) and alternatively activated macrophages (anti-inflammatory or M1 macrophages) (Marakalala et al., 2018; Khan et al., 2019). Macrophages with M1-like phenotype secret pro-inflammatory cytokines such as IL-1, TNF-α, IL-6, etc. and have powerful abilities to eradicate infecting mycobacteria. Whereas, macrophages with an M2-like phenotype secrete immunosuppressive cytokines such as IL-4 and IL-10, which are more permissive to the growth of *Mtb* (Khan et al., 2019). Therefore, the phenotypes of macrophages involved in *Mtb* infection might play a central role in determining the infection progresses (Khan et al., 2019). *Mtb* could alter the activation state of recruited macrophages and polarize macrophages *via* the induction of cytokines and chemokine they produce for their own benefit (Khan et al., 2019). Our research illustrated that FBXW7 is involved in the formation of tubercular lesions. In this study, we found that FBXW7 induced by wild *Mm* was associated with an increase in the anti-inflammatory cytokines including TGF-β and IL-10, but restrained the induction of pro-inflammatory mediators such as TNF-α and iNOS. This result indicated that FBXW7 may promote macrophages to differentiate toward an anti-inflammatory (M2-like) macrophage activation program. Our data corroborated the previous reports that *Mtb*-infected macrophages are predisposed to differentiate into M2 phenotype which exhibit the properties of enhanced protease-dependent motility, pathogen survival, and immuno-modulation (Lastrucci et al., 2015; Refai et al., 2018; Khan et al., 2019). Until now, very little is known about the mechanisms that *Mtb* alters in the macrophages phenotype. One perspective is that virulent *Mtb* utilized IL-1 receptor-associated kinase restricted to monocytic cells (IRAK-M) to restrain TLR-mediated NF-κB activation by limiting the activation of TNF˗α receptor associated factor (TRAF), and this mechanism pushed macrophage polarity toward an M2 phenotype to facilitate the organisms’ intracellular survival (Lastrucci et al., 2015; Shen et al., 2017; Khan et al., 2019). Another study reported that the Early Secreted Antigenic Target protein-6 (ESAT-6) derived from *Mtb* directly inhibited the activation of NF-κB and IFN regulatory factors (IRFs) downstream of TLR2 signaling *via* Akt-dependent mechanisms (Pathak et al., 2007; Refai et al., 2018). Consistent with these findings, our study showed that NF-𝜅Bp65 was also inhibited in the wild *Mm*-infected Raw264.7 cells. The expression NF-κBp65 was up-regulated and translocated to the nucleus of cells when FBXW7 was silenced in the *Mm*-infected cells, indicating that *Mm* may alter the phenotype of macrophages into anti-inflammatory phenotype *via* overexpression of FBXW7. However, more evidence is needed to define what other molecules are involved in this pathway. Using zebrafish as the infection model to visualize the dynamics of these macrophages in the context of FBXW7 expression could contribute to understanding the role of FBXW7 on differentiation of macrophages.

Many cytokines are involved in the development of granulomas during the *Mtb* infection. TNF-α is a crucial pro-inflammatory cytokine in the protective immune responses of host responsive to *Mtb* (Flynn et al., 1995; Dorhoi and Kaufmann, 2014), and a considerable concentration of TNF-α is necessary for the formation of *Mtb*-induced granulomas. A recent study about *Salmonella*’s role in macrophage polarization demonstrated that TNF signaling limited M2 granuloma macrophage polarization, thereby restricting the intracellular bacteria persistence (Pham et al., 2020). In the early stage of *Mtb* infection, host cells secrete TNF-α to activate macrophages and then activate T cell-mediated immune responses to mycobacteria (Li et al., 2020). However, it was found that *Mtb* could inhibit the secretion of TNF-α through bacterial substituents like PGL (Reed et al., 2004) and PtpA (Wang et al., 2015). It was recently revealed that *Mtb* manipulates the host to inhibit pro-inflammatory cytokine production such as TNF-α by disturbing the ubiquitin system components TAB3 and ANAPC2 (Wang L. et al., 2020). This is the first report that mycobacteria up-regulate FBXW7 in the host contributing to modulating the induction of TNF-α. In our study, TNF-α expression was recovered in both macrophages and tail tissues when FBXW7 expression was partial inhibited with a protease inhibitor, indicating that the expression of TNF-α was restrained by FBXW7. However, the mechanism of FBXW7 regulating the secretion of TNF-α is still unknown. It was reported FBXW7 targets P100, which restrained the activation of NF-κB pathway (Busino et al., 2012). Therefore, we hypothesized that the NF-κB signaling pathway would be active when FBXW7 was upregulated during *Mm* infection. Contrary to the speculation, we found that FBXW7 downregulated the expression of NF-κBp65. One explanation is that there are other substrate(s) regulating the activation of NF-κB signaling pathway besides of P100 during the infection. Another possibility is that the NF-κB signaling pathway was insufficiently activated under the microenvironment of lower production of TNF-α.

It was further shown that FBXW7 changed the ubiquitination level of proteins in macrophages, mainly K63-linked ubiquitination. This indicated that the degradation of TNF-α induced by FBXW7 might be associated with K63-linked ubiquitination. Further exploration is needed to determine whether FBXW7 regulates TNF-α directly or by interacting with another substrate(s). Moreover, there is a seemingly contradictory point between the negative regulation of FBXW7 on TNF-α vs FBXW7-mediated granulomas formation in this study.

The effects of *Mtb* on the host immune responses are multidirectional and complex. It has been identified that the virulent strain *Mtb* induced fewer TNF-α than the attenuated strains such as *M. smegmatis* and *M. phle*. Reduced TNF-α contributes to a more persistent intracellular infection in the hosts (Beltan et al., 2000; Olsen et al., 2016; Yao et al., 2018; Wang L. et al., 2020). Our study was consistent with these previous data. Overall, we demonstrated that FBXW7 plays an important role in the formation of the *Mm* tail granulomas, which is a crucial event in the pathogenesis of *Mtb*. It promotes the polarization of macrophages into the M2 phenotype and modulates TNF-α expression through a ubiquitination pathway. These data indicated that FBXW7 may be developed as a potential host-directed therapeutic target for TB treatment. However, a more comprehensive understanding of FBXW7 is required, such as identifying the components of *Mtb* that regulate the expression of FBXW7, the interaction between FBXW7 and TNF-α or other molecules and substrates, and so on.

## Data Availability Statement

The original contributions presented in the study are included in the article/**Supplementary Material**. Further inquiries can be directed to the corresponding authors.

## Ethics Statement

The animal study was reviewed and approved by Animal Care and Use Committee of CTGU.

## Author Contributions

DW and JS designed the project; JS, XW, XH, CD, DL, DZ, SH, XY, ZJ, YS, and JG performed the experiment; DW, JC, BY, LZ, and LV analyzed the data and wrote the draft; JC, LV, and DW finished the revision of this work. All authors contributed to the article and approved the submitted version.

## Funding

This work was supported in part by the National Natural Science Foundation of China (NSFC Nos. 31772709 and 31572485 to DW), the National Key Research and Development Program of China (2021YFC2301500 to LZ) a research project of the Science and Technology Bureau of Yichang (A21-2-049 to JC), the new faculty startup research funds of China Three Gorges University (KJ2014B023 to DW, 8210403 to JC), and the Scientific Research Project of Education Department of Hubei Province (B2020025 to SH). BY is the recipient of NSFC (81801977). LV and JG are supported by the intramural research program of NIAID, NIH.

## Conflict of Interest

The authors declare that the research was conducted in the absence of any commercial or financial relationships that could be construed as a potential conflict of interest.

## Publisher’s Note

All claims expressed in this article are solely those of the authors and do not necessarily represent those of their affiliated organizations, or those of the publisher, the editors and the reviewers. Any product that may be evaluated in this article, or claim that may be made by its manufacturer, is not guaranteed or endorsed by the publisher.

## Supplementary Material

Supplementary Table 1 Sequences the primers applied in quantitative real-time reverse transcriptase PCR.

Supplementary Figure 1 TGF-β and IL-10 expression in protein level when FBXW7 was silenced in Raw264.7 cells. **(A)** FBXW7 was silenced in Raw264.7 cells with small interfering RNA; **(B)** TGF-β and IL-10 expression were detected with Western blot.

Supplementary Figure 2 FBXW7 was overexpressed with CRISPR/Cas9 in Raw264.7.

Supplementary Presentation 1 Publishing Agreement and Manuscript Cover Sheet.
