# Supplementary material for: E3 Ligase FBXW7 Facilitates Mycobacterium Immune Evasion by Modulating TNF-α Expression
Source: Front Cell Infect Microbiol. 2022 May 16;12:851197. doi: 10.3389/fcimb.2022.851197 (PMC9149249; doi:10.3389/fcimb.2022.851197)
Supplement: Supplementary Table 1 — Sequences the primers applied in quantitative real-time reverse transcriptase PCR. [file Table_1.docx]

| Gene name | Genes sequence version | Forward primes | Reverse primes |
| --- | --- | --- | --- |
| Fbxw7 | NM_080428.3 | 5'-AGAGAAGTTGCTGGCTTTAGAT-3 | 5'-GCCAAAATTCTCCAGTATCGAC-3' |
| TNF-a | NM_013693.3 | 5’-TCAACCTCCTCTGCGAAG-3' | 5'-GAGCAATGACTCCAAAGTAGACCTG-3’ |
| TGF-β | XM_036152883.1 | 5'-TGCGCTTGCAGAGATTAAAA-3’ | 5’-AGCCCTGTATTCCGTCTTCT-3' |
| IL-10 | NM_010548.2 | 5'-ACCTGGTAGAAGTGATGCCC-3’ | 5’-ACACCTTGGTCTTGGAGCTT-3’ |
| iNOS | NM_001313922.1 | 5'-GCCCTGCTTTGTGCGAAG-3' | 5’-GCCCTTTGTGCTGGGAGTC-3' |
| GAPDH | NM_001289726.1 | 5'-AGTGTTTCCTCGTCCCGTAG-3’ | 5'-GCCGTGAGTGGAGTCATACT-3' |
