# Supplementary figures and images for: E3 Ligase FBXW7 Facilitates Mycobacterium Immune Evasion by Modulating TNF-α Expression
Source: Front Cell Infect Microbiol. 2022 May 16;12:851197. doi: 10.3389/fcimb.2022.851197 (PMC9149249; doi:10.3389/fcimb.2022.851197)

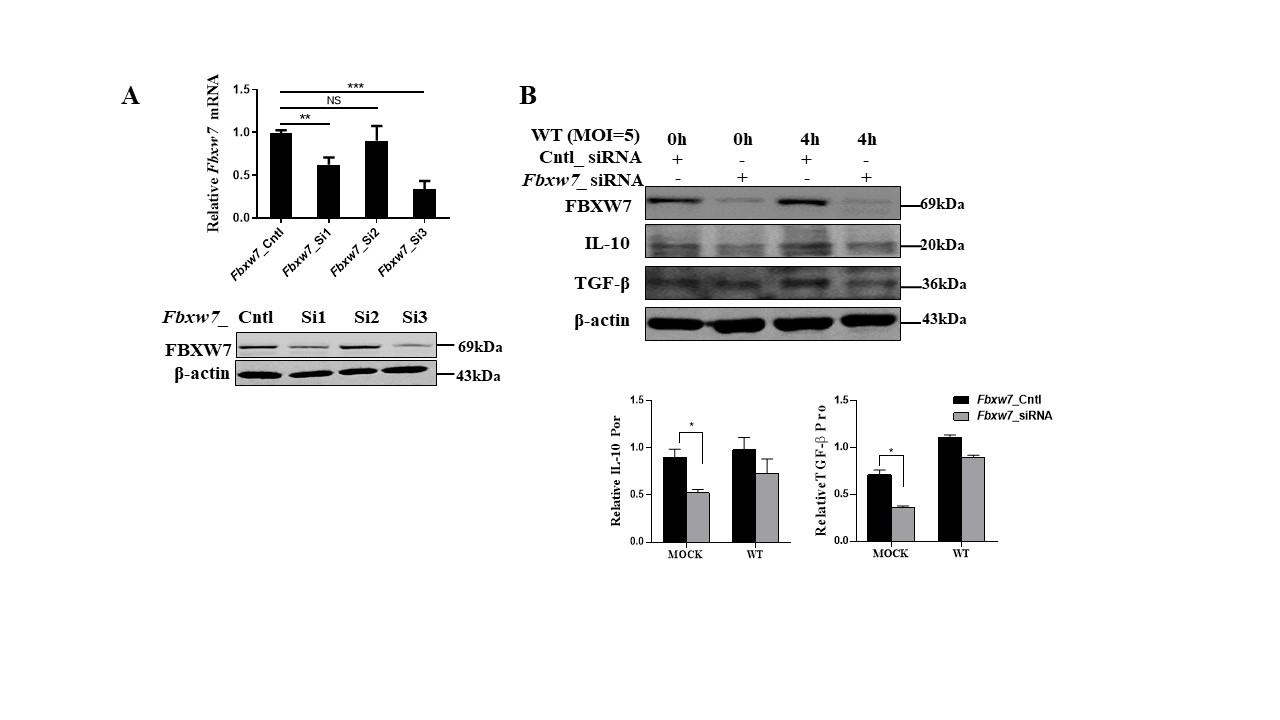

Supplement: Supplementary Figure 1 — TGF-β and IL-10 expression in protein level when FBXW7 was silenced in Raw264.7 cells. (A) FBXW7 was silenced in Raw264.7 cells with small interfering RNA; (B) TGF-β and IL-10 expression were detected with Western blot. [file Image_1.jpeg]

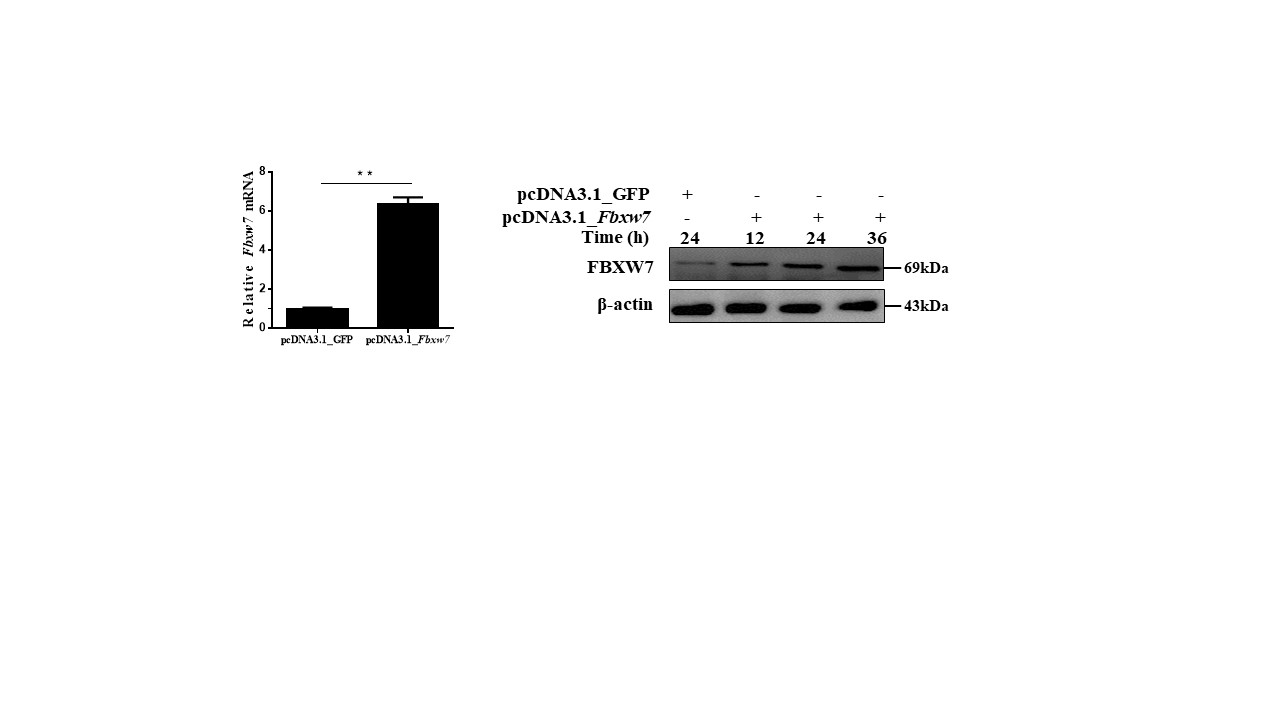

Supplement: Supplementary Figure 2 — FBXW7 was overexpressed with CRISPR/Cas9 in Raw264.7. [file Image_2.jpeg]
