# Supplementary material for: E3 Ligase FBXW7 Facilitates Mycobacterium Immune Evasion by Modulating TNF-α Expression
Source: Front Cell Infect Microbiol. 2022 May 16;12:851197. doi: 10.3389/fcimb.2022.851197 (PMC9149249; doi:10.3389/fcimb.2022.851197)
Supplement: Supplementary Presentation 1 — Publishing Agreement and Manuscript Cover Sheet. [file Table_2.docx]

| siRNA | Forward primes | Reverse primes |
| --- | --- | --- |
| Fbxw7-mus-s1 | 53-GCUCAGACUUGUCGAUACUTT-3’ | 5'-AGUAUCGACAAGUCUGAGCTT-3' |
| Fbxw7-mus-s2 | 53-CCUGCUUACAGUUCAACAATT-3’ | 5-UUGUUGAACUGUAAGCAGGTT-3' |
| Fbxw7-mus-s3 | 5-GGUUGUUAGUGGAGCUUAUTT-3’ | 5'-AUAAGCUCCACUAACAACCTT-3' |
| Negative control | 5'-UUCUCCGAACGUGUCACGUTT-3' | 5'-ACGUGACACGUUCGGAGAATT-3' |
